# DYRK1A Controls HIV-1 Replication at a Transcriptional Level in an NFAT Dependent Manner

**DOI:** 10.1371/journal.pone.0144229

**Published:** 2015-12-07

**Authors:** Thijs Booiman, Vladimir V. Loukachov, Karel A. van Dort, Angélique B. van ’t Wout, Neeltje A. Kootstra

**Affiliations:** Department of Experimental Immunology, Sanquin Research, Landsteiner Laboratory and Center for Infection and Immunity (CINIMA) at the Academic Medical Center of the University of Amsterdam, Amsterdam, The Netherlands; International Centre for Genetic Engineering and Biotechnology, ITALY

## Abstract

**Background:**

Transcription of the HIV-1 provirus is regulated by both viral and host proteins and is very important in the context of viral latency. In latently infected cells, viral gene expression is inhibited as a result of the sequestration of host transcription factors and epigenetic modifications.

**Results:**

In our present study we analyzed the effect of host factor dual specificity tyrosine-phosphorylation-regulated kinase 1A (DYRK1A) on HIV-1 replication. We show that DYRK1A controls HIV-1 replication by regulating provirus transcription. Downregulation or inhibition of DYRK1A increased LTR-driven transcription and viral replication in cell lines and primary PBMC. Furthermore, inhibition of DYRK1A resulted in reactivation of latent HIV-1 provirus to a similar extent as two commonly used broad-spectrum HDAC inhibitors. We observed that DYRK1A regulates HIV-1 transcription via the Nuclear Factor of Activated T-cells (NFAT) by promoting its translocation from the nucleus to the cytoplasm. Therefore, inhibition of DYRK1A results in increased nuclear levels of NFAT and increased NFAT binding to the viral LTR and thus increasing viral transcription.

**Conclusions:**

Our data indicate that host factor DYRK1A plays a role in the regulation of viral transcription and latency. Therefore, DYRK1A might be an attractive candidate for therapeutic strategies targeting the viral reservoir.

## Background

The ability of the human immunodeficiency virus type 1 (HIV-1) to replicate in a host cell is influenced by numerous host factors that act on different steps of the viral life cycle ranging from virus entry to budding of the newly formed virions. Recent genome wide RNAi studies have identified almost 1000 host proteins that support HIV-1 replication [1–9]. On the other hand, a number of host factors, such as MX2 [10–12], TRIM5α [13,14], SAMHD1 [15,16], APOBEC3 [17–19] and Tetherin [20] have been described to display antiviral effects and restrict viral replication.

Recently, we have performed a genome wide association study to assess the effect of genetic polymorphisms on HIV-1 replication in macrophages and we identified polymorphisms in a number of host genes that were strongly associated with HIV-1 replication [21]. One of these polymorphisms was located in the *dual specificity tyrosine-phosphorylation-regulated kinase 1A* (*DYRK1A*). In addition, this polymorphism was also associated with HIV-1 disease progression in two independent cohorts, suggesting an important role for this protein in HIV-1 replication [21].

DYRK1A is a kinase that is involved in regulation of the cell cycle and neurogenesis during brain development [22–27]. DYRK1A regulates the activity of several transcription factors [28–35], some of which have been implicated in the regulation of HIV-1 transcription [36–39]. DYRK1A phosphorylates the Nuclear Factor of Activated T-cells (NFAT) and the class III histone deacetylase Sirtuin 1 (SIRT1) [34,35]. Phosphorylation of NFAT by DYRK1A results in its translocation from the nucleus to the cytoplasm, which decreases nuclear NFAT levels [32,33]. SIRT1 phosphorylation by DYRK1A results in the activation of SIRT1, which deacetylates the RelA/p65 subunit of nuclear factor kappa-light-chain-enhancer of activated B cells (NF-κB) complex, and thus inhibits NF-kB activity [34]. Both NFAT and NF-kB are transcription factors that bind to the HIV-1 long terminal repeat (LTR) promoter thereby regulating proviral transcription [36–41].

Here we investigated the role of DYRK1A in HIV-1 replication. We show that DYRK1A controls HIV-1 replication at a transcriptional level in multiple cell lines and primary PBMC. DYRK1A inhibits LTR-driven transcription by limiting the nuclear localization of transcription factor NFAT. Inhibition of DYRK1A in TZM-bl cells and J-Lat cells, which carry a latent HIV-1 provirus, resulted in reactivation of the latent HIV-1 to a similar extent as treatment with TNFα and two commonly used broad-spectrum HDAC inhibitors. These data suggest that DYRK1A can control HIV-1 replication and might be involved in viral latency.

## Results

### DYRK1A knockdown or inhibition increases HIV-1 replication

**Fig 1:**
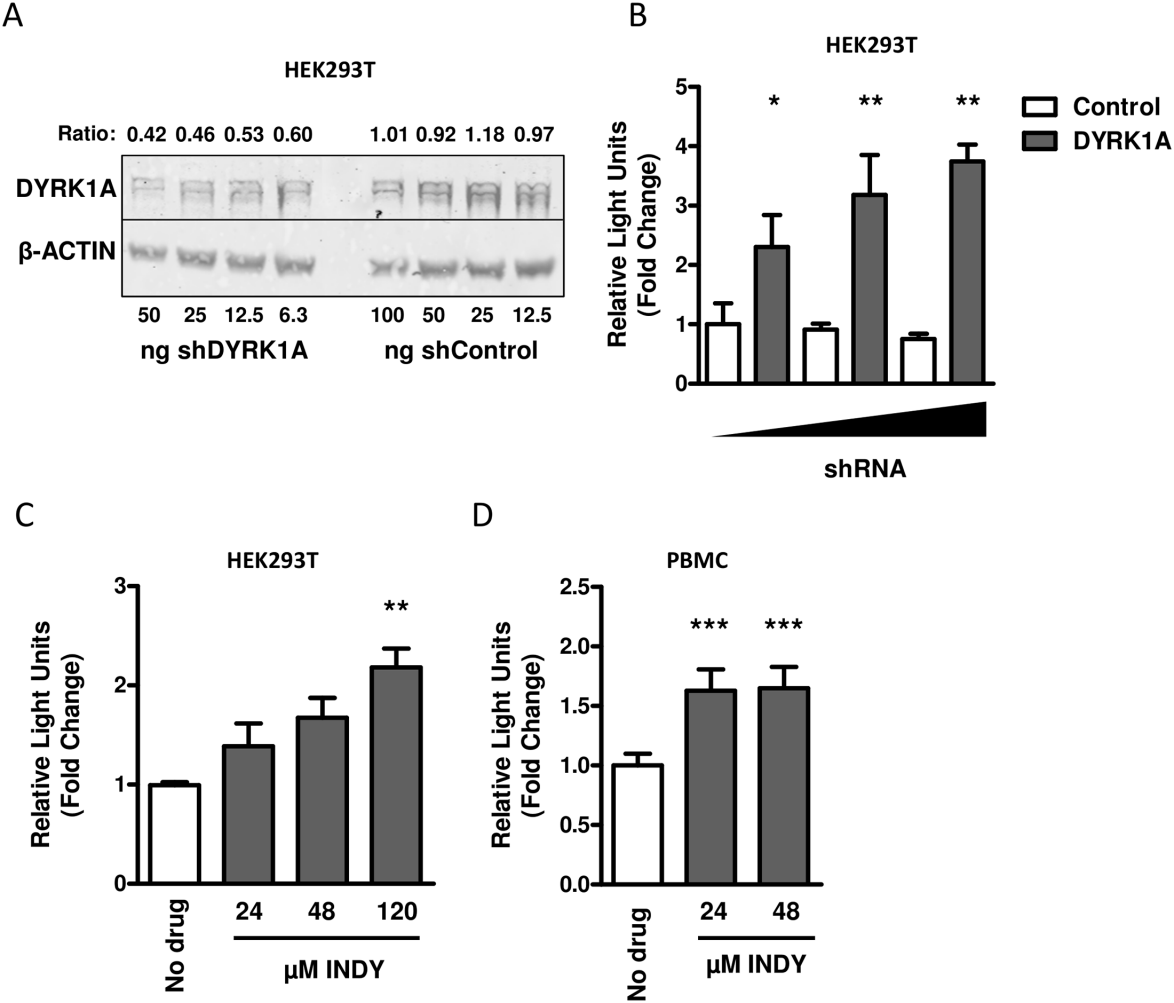
The effect of DYRK1A knockdown or inhibition on HIV-1 replication. (A) To test the shRNA targeting against DYRK1A, HEK293T cells were transfected with either 6.25 ng, 12.5 ng or 25 ng of shDYRK1A or shControl and 48-hours after transfection DYRK1A protein expression was determined by Western blot. The results shown are representative for 3 independent experiments. The ratio is calculated by dividing the intensity of the DYRK1A bands by the intensity of the β-actin band as determined with ImageJ. (B) The effect of DYRK1A downregulation on HIV-1 replication was tested by transfecting HEK293T cells in 96-wells plates with either 6.25 ng, 12.5 ng or 25 ng of shDYRK1A or corresponding concentrations of the shControl. Forty-eight hours after DYRK1A downregulation cells were inoculated at a MOI of 0.01 with a VSV-G-pseudotyped single-round luciferase virus. Luciferase activity was determined 48 hours post infection as a measure for viral replication and expressed relative to the corresponding shControl. Data is shown as mean and SD of three independent experiments. (C) The effect of DYRK1A inhibition on HIV-1 replication in HEK293T was analyzed by infection with a VSV-G-pseudotyped single-round luciferase virus. Twenty-four hours post infection cells were treated with 24 μM, 48 μM or 120 μM of either INDY or DMSO control and after an additional 24-hours luciferase activity was determined as a measure for viral replication and expressed relative to DMSO control (No Drug). Data is shown as mean and SD of three independent experiments. (D) The effect of DYRK1A inhibition on HIV-1 replication in PBMC was analyzed by infecting PHA-stimulated PBMC from four healthy blood donors with a VSV-G-pseudotyped single-round luciferase virus at a MOI of 0.1. Twenty-four hours post infection, cells were treated with either 24 μM or 48 μM of INDY or DMSO control and after another 24-hours luciferase activity was determined as a measure for viral replication and expressed relative to the DMSO control (No drug). Data is shown as mean and SD of 4 independent donors. Significance was determined with an unpaired student’s T test. *p<0.05, **p<0.01, ***p<0.001.

The effect of DYKR1A knockdown on HIV-1 replication was analyzed in HEK293T cells. HEK293T cells express high levels of endogenous DYRK1A and after transfection with a shRNA that targets DYRK1A mRNA, a dose-dependent decrease in DYRK1A protein expression was observed (Fig 1A). When the DYRK1A knockdown cells were infected with a VSV-G pseudotyped HIV-1 luciferase reporter virus, a dose dependent increase in luciferase activity was observed (Fig 1B and S1A Fig). This indicates that DYRK1A represses viral replication in HEK293T cells. A similar observation on virus replication was made using INDY, a selective inhibitor of DYRK1A [42,43]. When INDY was added to the HEK293T cells at 24 hours after infection with a VSV-G pseudotyped HIV-1 luciferase reporter virus, a dose dependent increase in luciferase activity was observed (Fig 1C and S1B Fig). Next, we analyzed whether DYKR1A is also an important regulator of HIV-1 replication in primary cells. Activated PBMCs were infected with a VSV-G pseudotyped HIV-1 luciferase reporter virus and 24-hours after infection different concentrations of the DYRK1A inhibitor INDY were added to the culture medium. After 24-hours, we observed an increase in luciferase activity in the PBMC that were treated with the DYRK1A inhibitor (Fig 1D). This indicates that DYRK1A also controls HIV-1 replication in PBMC.

### DYRK1A affects HIV-1 replication at a transcriptional level

**Fig 2:**
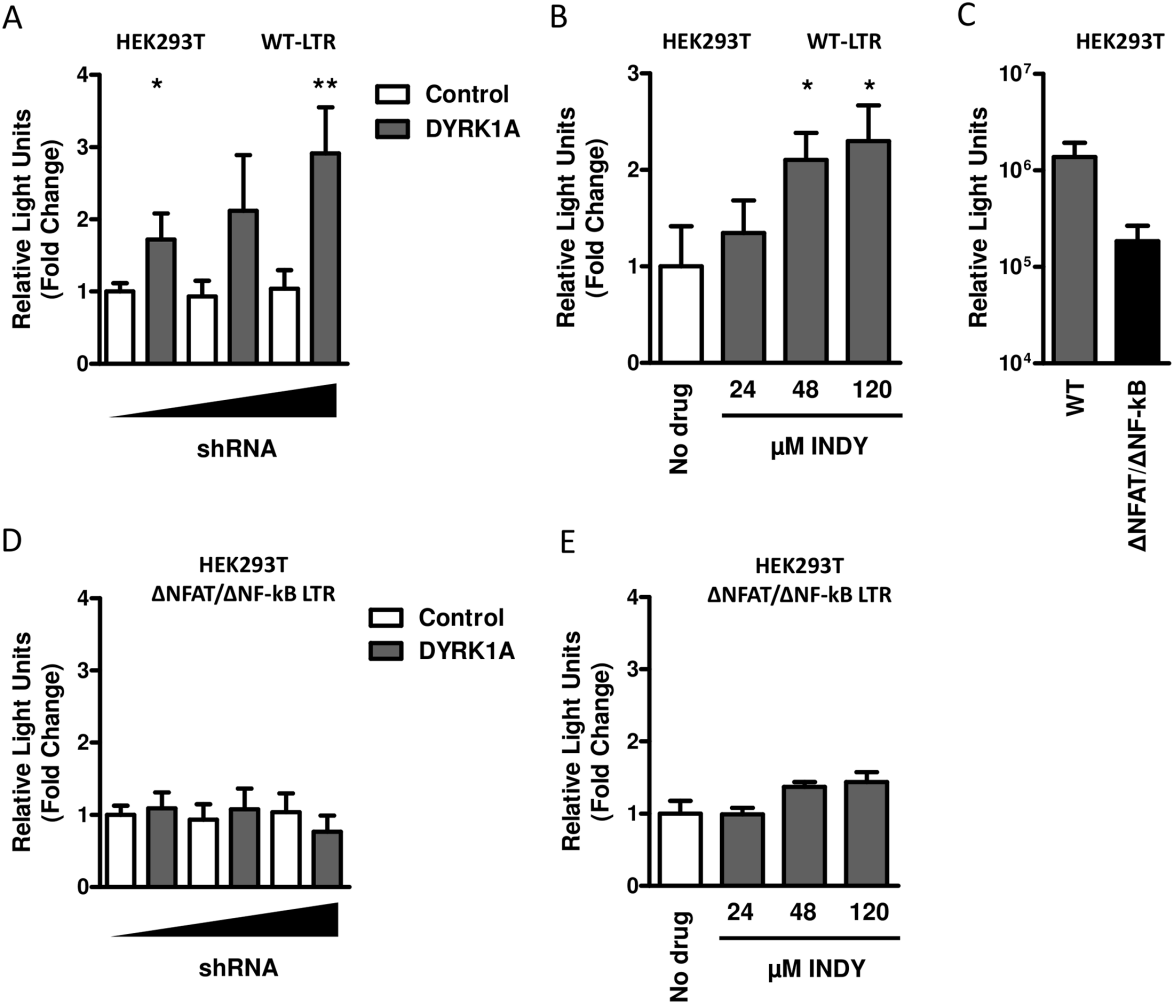
The effect of DYRK1A knockdown or inhibition on LTR driven transcription. (A) The effect of DYRK1A downregulation on LTR driven transcription was analyzed by co-transfection of HEK293T cells in 96-wells plates with 5 ng of LTR-luciferase reporter construct and 12.5 ng, 25 ng or 50 ng of shDYRK1A or the shControl vector. Luciferase activity was analyzed 48-hours post-transfection as a measure for LTR activity and expressed relative to the shControl. (B) The effect of DYRK1A inhibition on LTR driven transcription was analyzed by transfecting HEK293T cells in 96-wells plates with 5 ng of LTR-luciferase reporter construct. Twenty-four hours post transfection cells were treated with 24 μM, 48 μM or 120 μM of either INDY or the DMSO control and after an additional 24-hours luciferase activity was determined and expressed relative to DMSO control (No Drug). (C) Comparison of basal transcriptional levels of the LTR-luciferase reporter construct and LTR-luciferase reporter construct lacking the NFAT and NF-kB binding sites was performed by transfecting HEK293T cells in 96-wells plates with 5 ng of either construct. Luciferase activity was analyzed 48-hours post-transfection as a measure for LTR activity. The effect of DYRK1A downregulation (D) or inhibition (E) on LTR driven transcription was analyzed by co-transfection of HEK293T cells in 96-wells plates with 5 ng of LTR-luciferase reporter construct lacking the NFAT and NF-kB binding sites and 12.5 ng, 25 ng or 50 ng of shDYRK1A or the shControl vector or treatment with 24 μM, 48 μM or 120 μM INDY. Luciferase activity was analyzed 48-hours post-transfection as a measure for LTR activity and expressed relative to the shControl. Significance was determined with an unpaired student’s T test. *p<0.05, **p<0.01. Data is shown as mean and SD of three independent experiments.

DYRK1A has been described to regulate the activity of several transcription factors, including NFAT and NF-kB, which are known to drive transcription from the HIV-1 LTR [36–39]. We investigated whether the effect of DYRK1A on HIV-1 replication was mediated at a transcriptional level. We co-transfected reporter constructs in which luciferase expression is driven by the HIV-1 LTR in combination with a shRNA against DYRK1A in HEK293T cells and analyzed luciferase activity 48-hours after transfection as a measure for LTR driven transcription. A dose dependent increase in luciferase activity was observed in HEK293T cells in which DYRK1A expression was downregulated by shRNA (Fig 2A). Moreover, a dose dependent increase of LTR driven luciferase expression was observed when DYRK1A was inhibited by increasing concentrations of INDY (Fig 2B). Next, we analyzed whether DYRK1A regulates viral transcription via the transcription factors NFAT and NF-kB by using a luciferase reporter construct in which the NFAT an NF-kB binding sites were deleted from the LTR. Although the level of basal transcription of this construct is decreased compared to the construct containing the complete LTR, significant levels of luciferase activity could be detected (Fig 2C). Knockdown or inhibition of DYRK1A did not affect transcription driven by LTR from which the NFAT and NF-kB binding sites were removed (Fig 2D and 2E). This suggests that DYRK1A controls HIV-1 replication by repressing transcription most likely via the nuclear factors NFAT and/or NF-kB.

### Inhibition of DYRK1A reactivates HIV-1 transcription

**Fig 3:**
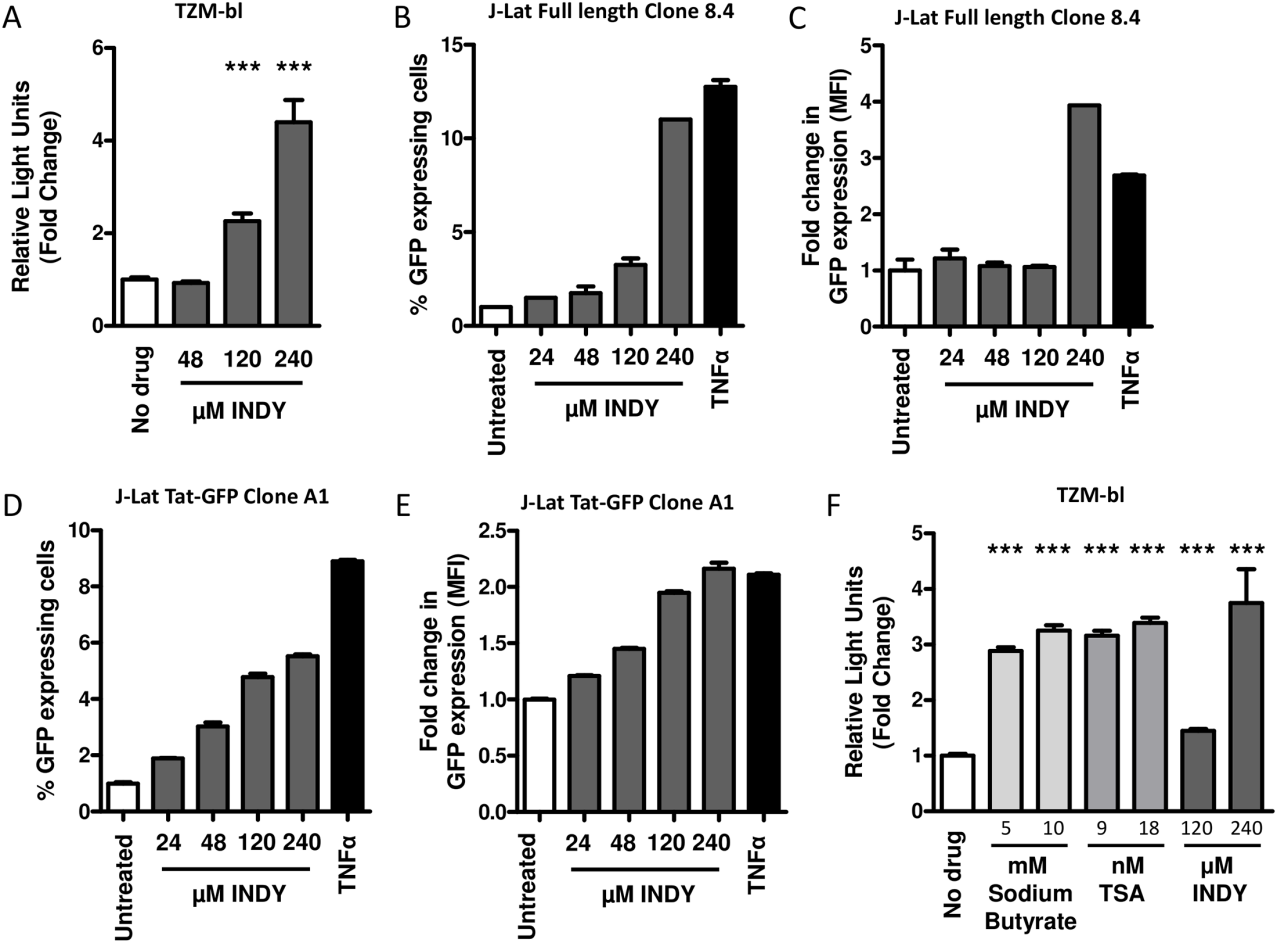
The effect of DYRK1A inhibition on reactivation of HIV-1 LTRs. (A) The effect of DYRK1A inhibition on reactivation of silent HIV-1 provirus was studied in TZM-bl cells. TZM-bl were treated with either 48 μM, 120 μM or 240 μM of INDY or the DMSO control and 24-hours later LTR-driven luciferase activity was determined as a measure for viral reactivation. Results are expressed relative to the DMSO control. Data is shown as mean and SD of three independent experiments. (B-E) J-Lat cells were treated for twenty four hours with 24, μM, 48 μM, 120 μM or 240 μM of INDY or 12.5 ng/ml TNFα as a positive control. Subsequently the percentage of GFP expressing cells and the mean fluorescent intensity was determined by FACS. Results are expressed relative to the control). Data is shown as mean and SD of two independent experiments. (F) The effect of DYRK1A inhibition on the reactivation of silent HIV-1 provirus was compared to reactivation by two HDAC inhibitors sodium butyrate and TSA. TZM-bl were treated with either 120 μM or 240 μM of INDY, 5 mM or 10 mM of Sodium butyrate, or 9 nM or 18 nM of TSA or the appropriate vehicle control. Twenty-four hours later, LTR-driven luciferase activity was determined and expressed relative to vehicle control. Data is shown as mean and SD of three independent experiments. Significance was determined with an unpaired student’s T test. *p<0.05, **p<0.01, ***p<0.001.

To analyze whether DYRK1A plays a role in transcriptional silencing of an integrated HIV-1 provirus, we analyzed whether the DYRK1A inhibitor INDY is able to reactivate a transcriptionally latent HIV-1 provirus. As model systems we used TZM-bl and J-Lat cells which contain an integrated luciferase or GFP gene under the control of the HIV-1 LTR promoter. When TZM-bl cells were incubated with different concentrations of the DYRK1A inhibitor INDY, we observed that luciferase expression strongly increased, indicating that inhibition of DYRK1A resulted in reactivation of the HIV-1 LTR (Fig 3A and S1C Fig). Similar results were observed when J-Lat cells were treated with INDY [44,45]. Treatment of J-Lat full length cells (8.2) and J-Lat TAT-GFP cells (A1) resulted in increased GFP expression and also increased numbers of GFP expressing cells (Fig 3B–3E). For comparison, we analyzed the ability of two broad spectrum histone deacetylase inhibitors (HDACi’s), sodium butyrate and trichostatin A (TSA) [46–51], to reactivate transcription from the HIV-1 LTR. Increased luciferase activity was indeed observed in the presence of these HDACi. Inhibition of DYRK1A resulted in reactivation of the latent provirus to a similar extent as the two broad spectrum HDACi’s tested (Fig 3E and S1C Fig).

### The effect of DYRK1A on HIV-1 transcription and replication is mediated via transcription factor NFAT

**Fig 4:**
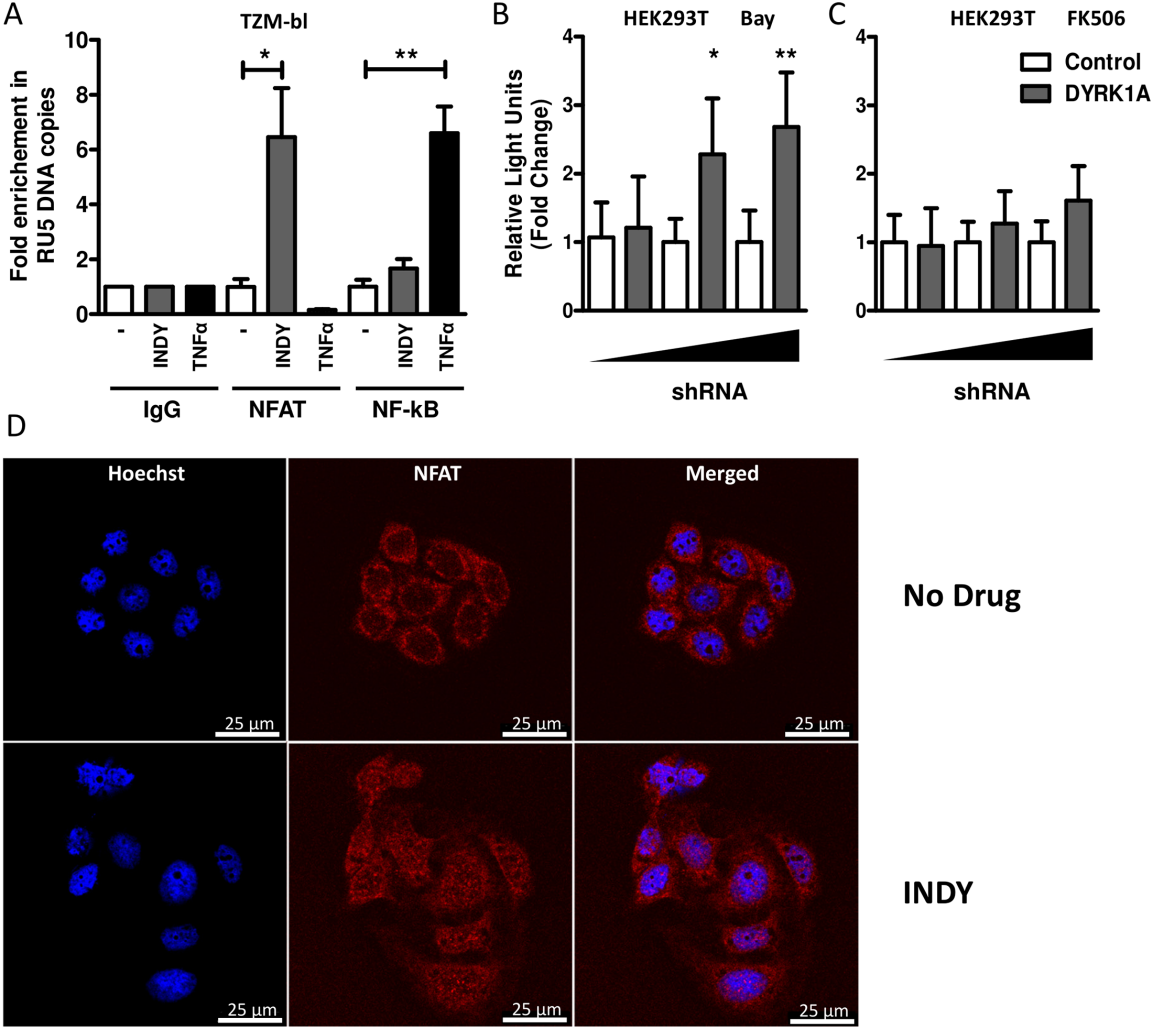
The effect of DYRK1A inhibition and downregulation is mediated via NFAT. (A) To analyze the effect of DYRK1A inhibition on the amount of NFAT or NF-kB bound to the viral LTR, a ChIP-qPCR analysis was performed in TZM-bl cells treated with either 240 μM of INDY, the DMSO control, or 12.5 ng/ml TNFα. Sheared DNA was immunoprecipitated with either control IgG, anti-NFAT, or anti-NF-κB antibodies and levels of bound LTR DNA were analyzed by qPCR. (B) The effect of DYRK1A downregulation on LTR driven transcription in the presence of 10μM NF-kB inhibitor BAY or 300 ng/ml NFAT inhibitor FK506 (C) was analyzed by co-transfection of HEK293T cells in 96-wells plates with 5 ng of LTR-luciferase reporter construct and 12.5 ng, 25 ng or 50 ng of shDYRK1A or the shControl vector. Luciferase activity was analyzed 48-hours post-transfection as a measure for LTR activity and expressed relative to the shControl. Data is shown as mean and SD of three independent experiments. (D) Nuclear localization of NFAT was studied in TZM-bl cells cultured for 24 hours in the absence or presence of 240 μM INDY. Subsequently, cells were stained with Hoechst and anti-NFAT and analysed by confocal fluorescent microscopy. Results are representative of at least two independent experiments. Significance was determined with an unpaired student’s T test. *p<0.05, **p<0.01, ***p<0.001.

Our data suggests that DYRK1A regulates transcription from the viral LTR through the transcription factors NFAT and/or NF-kB. To confirm this, we performed DNA chromatin immunoprecipitations in TZM-bl cells cultured in the presence or absence of INDY. Treatment with INDY increased binding of NFAT to the HIV-1 LTR, whereas binding of NF-kB to the HIV-1 LTR only increased upon TNFα treatment (Fig 4A). To further show that the effect of DYRK1A on HIV-1 replication and transcription is mediated via NFAT we tested whether inhibition of NFAT or NF-kB would abrogate the effect of DYRK1A knockdown on LTR-driven transcription in HEK293T cells. Indeed, treatment of HEK293T with NFAT inhibitor FK506 abrogated the effect of DYRK1A knock-down, whereas treatment with NF-kB inhibitor Bay had no effect (Fig 4B, 4C and S1B Fig). Of note, both inhibitors were effective at the concentration used as demonstrated by the decrease of the basal activity of the LTR-luciferase reporter construct (S2A Fig). It has been shown before that DYRK1A can phosphorylate NFAT, thereby promoting its translocation from the nucleus to the cytosol [32,33]. The increase in HIV-1 transcription upon DYRK1A inhibition might therefore be the result of an increased nuclear localization and subsequent binding of NFAT to the HIV-1 LTR. We therefore analyzed NFAT translocation upon inhibition of DYRK1A by INDY by confocal microscopy. TZM-bl cells were treated with INDY and subsequently stained with Hoechst and an antibody against NFAT. Treatment with INDY resulted in a translocation of NFAT into the nucleus, which further confirms that DYRK1A affects HIV-1 transcription by regulating the nuclear localization of NFAT (Fig 4D).

## Discussion

Retroviral transcription is a complex process and is regulated by both viral and host proteins. In the present study we show that DYRK1A can control HIV-1 replication at a transcriptional level. Downregulation of DYRK1A expression by shRNA or inhibition by INDY increased viral replication at a transcriptional level. DYRK1A mediated inhibition was dependent of NFAT and/or NF-kB, since transcription using a LTR construct lacking the NFAT and NF-kB binding sites was not affected by DYRK1A. When we analyzed the amount of NFAT and NF-kB bound to the viral LTR, we observed that inhibition of DYRK1A induced recruitment of NFAT to the viral LTR. This was further supported by the finding that inhibition of NFAT but not NF-kB abrogated the effect of DYKR1A knock-down on LTR-driven transcription. DYRK1A is known to phosphorylate NFAT, which results in translocation of NFAT from the nucleus into the cytoplasm [32,33]. Indeed, we observed increased translocation of NFAT upon DYRK1A inhibition by INDY. Thus, our data show that DYRK1A inhibition or downregulation of DYRK1A expression results in higher nuclear NFAT levels and increased binding of NFAT to the HIV-1 LTR.

It has been suggested that the inactivation of RelA/p65 by SIRT1 can be prevented by the binding of the HIV-1 Tat protein to the deacetylation domain of SIRT1 [52,53]. However, our data suggests that the effect of DYRK1A is not mediated via SIRT1 and NF-kB (Fig 4B), and also co-transfection of TAT did not affect the increase in luciferase activity upon DYRK1A knock-down (S2B Fig).

Here we observed that DYRK1A is a potent regulator of viral transcription and acts through translocation of the transcription factor NFAT. Sequestration of transcription factors like NFAT has been shown to play a major role in the development of HIV-1 latency [54,55]. Interestingly, we observed that treatment of TZM-bl and J-Lat cells with DYRK1A inhibitor INDY resulted in activation of transcriptionally silent HIV-1 LTR promoters. The reactivation of proviral transcription by INDY was comparable to the reactivation achieved with two commonly used broad-spectrum HDAC inhibitors and TNFα. Reversal of viral latency in an attempt to purge the viral reservoir through killing of infected cells by cytolytic T cells or cytopathic effects of viral replication, using several HDAC inhibitors was successful to some extent [56–58]. However, it has been demonstrated that only a small proportion of latent proviruses can be reactivated with HDAC inhibitors [59,60]. This underscores the need to understand viral latency and cellular factors involved. The identification of cellular factors such as DYRK1A that regulate viral transcription will provide more insights in the complex process of viral transcription and might provide new therapeutic opportunities for drug development targeting the viral reservoir.

## Conclusions

DYRK1A controls HIV-1 replication at a transcriptional level and the effect of DYRK1A on provirus transcription is mediated by limiting the nuclear localization of transcription factor NFAT. Inhibition of DYRK1A resulted in the reactivation of latent integrated provirus, which indicates that DYRK1A is involved in the regulation of viral latency. Therefore, DYRK1A might be an attractive candidate for therapeutic strategies targeting the viral reservoir.

## Methods

### Ethics Statement

This study has been conducted in accordance with the ethical principles set out in the declaration of Helsinki, and was approved by the Medical Ethics Committee of the Academic Medical Center and the Ethics Advisory Body of the Sanquin Blood Supply Foundation in Amsterdam, The Netherlands. Written informed consent was obtained from all participants.

### Cell lines and virus production

HEK293T cells were cultured in Dulbecco's Modified Eagle Medium without Hepes (DMEM) (Lonza, Basel, Switzerland) supplemented with 10% (v/v) inactivated fetal calf serum (FCS), penicillin (100 U/ml) and streptomycin (100 μg/ml) [61,62]. TZM-bl cells were cultured in Iscove's modified Dulbecco medium supplemented with 10% fetal calf serum, penicillin (100 U/ml), streptomycin (100 U/ml) (Lonza) [63–67]. J-Lat cells were cultured in Roswell Park Memorial Institute Medium (RPMI) (Lonza) supplemented with 10% (v/v) heat-inactivated fetal calf serum (FCS), penicillin (100 U/ml) and streptomycin (100 μg/ml) [44,45]. All cells were maintained in a humidified 10% CO_2_ incubator at 37°C. VSV-G pseudotyped NL4-3.Luc.R-E- luciferase reporter virus was produced by transfection of pNL4-3.Luc.R-E- with pCMV-VSV-G in HEK293T cells [68,69]. Transfections were performed with the calcium phosphate method [70]. In short, plasmid DNA was diluted in 0.042M HEPES containing 0.15M CaCl_2_, subsequently mixed with an equal volume of 2× HEPES buffered saline pH 7.2, incubated at room temperature for 15 min and added to the culture medium. After 24 h incubation in a humidified 3% CO_2_ incubator at 37°C, the culture medium was replaced and cultures were continued at 10% CO_2_ at 37°C. Virus was harvested at 48 and 72 h after transfection and passed through a 0.22 μm filter. HIV-1 virus titers were quantified by determining the TCID50 on 293T cells [71].

### LTR-driven transcription in HEK293T, TZM-bl and J-Lat cells

HEK293T cells were transfected with pLKO.1 constructs expressing a shRNA against DYRK1A, a control shRNA (TRCN199464 or SHC001; Sigma-Aldrich, USA [72]) and/or the long terminal repeat (HXB2 LTR) luciferase reporter constructs pBlue3′ LTR-luc [73], and pBlue3′ LTRΔNFAT/ΔNF-kB-luc and/or HIV-1 Tat expression construct sv-Tat using the calcium phosphate method. Forty-eight hours after transfection, LTR-driven luciferase activity was analyzed. The effect of DYRK1A inhibition on LTR driven transcription was analyzed by transfecting HEK293T cells in 96-wells plates with 5 ng of LTR-luciferase reporter construct. Twenty-four hours post transfection cells were treated with 24 μM, 48 μM or 120 μM of either INDY (Glixx Laboratories Cat #:GLXC-02452, USA), or the DMSO control and/or 10 μM BAY 11–7082 (Calbiochem/MERCK Millipore, USA) or 300 ng/ml FK506 (Calbiochem/MERCK Millipore, USA). After an additional 24-hours luciferase activity was determined. To analyzed the effect of DYRK1A inhibition on reactivation of a silent HIV-1 LTR, TZM-bl were treated with either 48 μM, 120 μM, or 240 μM of INDY (Glixx), 5 mM or 10 mM of Sodium butyrate (Sigma-Aldrich), 9 nM or 18 nM of trichostatin A (TSA) (Sigma-Aldrich) or the appropriate vehicle control. Twenty-four hours later, LTR-driven luciferase activity was determined by using the luciferase activity reagent (LAR) containing 0.83 mM of ATP, 18.7 mM MgCl2, 0.78 μM Na2H2P2O7, 38.9 mM Tris (pH 7.8), 0.39% glycerol, 0.03% Triton x-100, 2.6 μM dithiothreitol and 0.83 mM of d-Luciferin (Duchefa Biochemie B.V., Haarlem, The Netherlands). 25 μl of LAR was added to the transfected cells and luminescence was immediately measured using a luminometer (Berthold Technologies, Germany).

J-Lat cells were cultured in the presence or absence of 24 μM, 48 μM, 120 μM or 240 μM INDY(Glixx) or 12.5 ng/ml TNFα (Peprotech, UK) as a positive control. Twenty-four after treatment cells medium was aspirated and cells were fixed with 1x BD CellFIX (BD biosciences, USA) and analysed for GFP expression with the FacsCanto II (BD biosciences), results were analyzed in FlowJo, version 9.4.3 (Tree Star, USA).

### Infection of HEK293T cells

HEK293T cells were transfected with pLKO.1 constructs expressing a shRNA against DYRK1A or a control shRNA. Forty-eight hours after transfection, cells were inoculated at a multiplicity of infection (MOI) of 0.01 with NL4-3 luciferase VSV-G-pseudotyped single-round reporter virus. Forty-eight hours after infection luciferase activity was analyzed as a measure for viral replication. To analyze the effect of DYRK1A inhibition on HIV-1 replication, HEK293T cells were inoculated at a multiplicity of infection (MOI) of 0.01 with NL4-3 luciferase VSV-G-pseudotyped single-round reporter virus. Twenty-four hours post infection cells were treated with 24 μM, 48 μM or 120 μM of either INDY (Glixx) or DMSO control and after an additional 24-hours luciferase activity was determined as a measure for viral replication.

### PBMC culture and infection

PBMC were obtained from buffy coats from healthy blood donors. Cells were isolated by density gradient centrifugation on Lymphoprep (Axis-Shield, Oslo, Norway) and were stimulated for 3 days in Iscove modified Dulbecco medium supplemented with 10% fetal bovine serum, penicillin (100 U/ml), streptomycin (100 U/ml), Ciproxin (5 μg/ml), and phytohemagglutinin (PHA; 5 μg/ml) at a cell concentration of 5 × 10^6^ per ml. After inoculation, the cells (10^6^/ml) were cultured in medium supplemented with 10% fetal bovine serum, penicillin (100 U/ml), streptomycin (100 U/ml), Ciproxin (5 μg/ml), recombinant interleukin-2 (20 U/ml; Chiron Benelux, Amsterdam, The Netherlands) and Polybrene (hexadimethrine bromide) (5 μg/ml; Sigma, Zwijndrecht, The Netherlands). PBMC stimulated with PHA were inoculated at a MOI of 0.1 with NL4-3 luciferase VSV-G-pseudotyped single-round reporter virus. After 24-hours specific DYRK1A inhibitor INDY (Glixx) or the DMSO control was added to the culture medium and after another 24-hours luciferase activity was analyzed as a measure for viral replication.

### MTT cell viability assay

Cell viability was determined by Thiazolyl blue tetrazolium blue (MTT) assay as described previously [74]. In brief, cells were incubated for 24 hours with the inhibitors. Subsequently, MTT (Sigma-Aldrich) was added to a final concentration of 0.5 mg/mL and cells were incubated for another 3 h in a humidified 5% CO2 incubator at 37°C. Next, medium was aspirated, 100 μL DMSO was added, and absorbance was measured at 580 nm. Background was determined by measuring absorption at 655 nm and subtracted from the measurement at 580 nm.

### Western blot analysis

The effect of DYRK1A knockdown by shRNAs on protein levels was analyzed by western blot. Two days post-transfection, HEK293T cells were lysed in RIPA-buffer (150 mM NaCl, 1% Triton X-100, 0.5% sodium deoxycholate, 0.1% SDS, 50 mM Tris, pH 8.0) containing Complete^®^ EDTA free protease inhibitor (Roche, Basel, Switzerland). After adding NuPAGE LDS 4x sample buffer (Invitrogen) and 0.1M DTT, samples were heated at 95°C for 10 min. The Odyssey Protein Weight Marker was used as a size reference (LI-COR, Lincoln, NE, USA). Proteins were separated by SDS-PAGE (NuPAGE 10% Bis-Tris precast gel and MES SDS running buffer (Invitrogen) and transferred to a nitrocellulose membrane (Protran, Schleicher & Schuell, Dassel, Germany) using NuPAGE transfer buffer. After blocking for 2 hours with PBS containing 5% Protifar (Nutricia, Schiphol, The Netherlands) and 0.5% bovine serum albumin, the blot was incubated with anti-DYRK1A antibody (1:200; H00001859-M01; Abnova, Taipei City, Taiwan) and anti-β-actin antibody (1:200; SC-1616; Santa Cruz Biotechnology, Santa Cruz, CA, USA). IRDye 800CW conjugated Goat anti-Mouse IgG (1:15000; 926–32210, LI-COR, Lincoln, NE, USA) and IRDye 680LT conjugated Donkey anti-Goat IgG (1:15000; 926–32224, LI-COR) were used as secondary antibodies to visualize expression using the Odyssey infrared image system (LI-COR). Image J software was used to quantify protein expression and DYRK1A expression was corrected by β-actin expression by taking the ration between DRYK1A and β-actin expression.

### Immunofluorescence Microscopy

TZM-bl cells were cultured onto 15 mm cover slips (MENZEL-GLÄSER Lot# 94711285, Germany) and treated with 240μM of INDY (Glixx) or the DMSO control. After twenty-four hours of incubation, cells were washed with PBS and fixated with 70% ice cold ethanol for 10 min. After fixation, the cells were washed with PBS and incubated with 5μg of anti-NFATc1 antibody (H-110: Santa Cruz, USA) for 30 min at 4°C. Next, cells were blocked for 30 min with PBS containing 0.5% bovine serum albumin. After a wash with PBS, cells were incubated with the secondary antibody: 1: 400 Donkey anti-Rabbit IgG (H+L) Secondary Antibody, Alexa Fluor^®^ 546 conjugate (#A10040, Invitrogen) and Hoechst 1:10,000 (H1398, Invitrogen) for 45 min at room temperature. Images were captured using a Leica confocal microscope TCS SP-8 X (Leica Microsystems, USA) and analyzed and processed using Leica Application Suite (Leica Microsystems).

### Chromatin immunoprecipitation

Chromatin immunoprecipitation (ChIP) assays were performed using the CHIP-IT Express Enzymatic kit (Active Motif, Cat # 53009,Carlsbad, California, USA) according to the manufacturers protocol. In short, TZM-bl cells were treated with either 240 μM INDY (Glixx), 12.ng/ml TNFα (Peprotech), or DMSO vehicle control in a 15 cm plate [63–67]. After 24-hours, cells were crosslinked with 1% formaldehyde for 10 min at room temperature before the reaction was stopped by adding glycine for 10 min at room temperature. Cells were removed from the plates with cells scrapers and provided cell-scrape solution. To release the nuclei, cells were lysed in the provided lysis buffer and incubated on ice for 30 min. Cell lysates were enzymatically sheared using the provided enzymatic shearing cocktail supplemented with 60 units of XbaI (Roche) and 30 units of NspI (New England Biolabs, Ipswich, Massachusetts, USA). The sheared lysates were immunoprecipitated overnight at 4°C with 2 μg of either the anti-NFATc1 antibody (H-110: Santa Cruz, California, USA), the anti-NF-κB p65 antibody (sc-372x, Santa Cruz, Biotechnology), or control mouse IgG (Active Motif, Cat # 53010) and 25μl Protein G Magnetic Beads. Beads were subsequently washed two times with 800 μl of ChIP buffer 1 and two times with 800 μl of ChIP buffer 2. Bound complexes were eluted by a 15 min incubation at room temperature in 50 μl of the provided elution buffer AM2. Chromatin was reverse cross-linked by addition of 50 μl of the provided reverse Cross-linking Buffer and incubation at 95°C for 15 min. Subsequently, 2 μl of the provided proteinase K was added and chromatin was incubated for 1 hour at 37°C. Next, the levels of HIV-1 LTR were quantified by qPCR with the following primers and probes: RU5-F 5’-GTGCCCGTCTGTTGTGTGAC-3’, RU5-R 5’-GGCGCCACTGCTAGAGATTT-3’ and RU5-P 5’-(FAM)-CTAGAGATCCCTCAGACCCTTTTAGTCAGTGTG-(TAMRA)-3’ [75]. DNA enrichment was calculated according to the manufacturer’s instructions: Fold enrichment = ChIP target DNA quantity / ChIP control IgG DNA quantity. qPCRs were performed on a LightCycler^®^ 2.0 (Roche) using the following program: pre-incubation steps of 2 min 50°C and 2 min 95°C and 45 amplification steps of 5s 94°C and 30s 60°C.

## Supporting Information

S1 Fig Effect of shRNAs (A) and inhibitors (B) on cell viability of HEK293T cells and (C) TZM-bl cells. Cell viability was assessed by MTT assay. Results are plotted as the mean and SD of at least two independent experiments and plotted as the fold change as compared to the untreated control cells.
(TIF)

S2 Fig (A) The effect of NF-kb inhibitor Bay and NFAT inhibitor FK506 on basal LTR-driven luciferase expression in HEK293T cells. HEK293T cells were transfected in 96-wells plates with 5 ng of LTR-luciferase reporter construct and treated with 10 μm Bay or 300 ng/ml FK506 24 hours post transfection. Luciferase activity was analyzed 48-hours post-transfection as a measure for LTR activity and expressed relative to the No drug control. Data is shown as mean and SD of three independent experiments. (B) The effect of DYRK1A downregulation on LTR driven transcription in the presence of HIV-Tat was analyzed by co-transfection of HEK293T cells in 96-wells plates with 5 ng of LTR-luciferase reporter construct, 5 ng SV-Tat and 12.5 ng, 25 ng or 50 ng of shDYRK1A or the shControl vector. Luciferase activity was analyzed 48-hours post-transfection as a measure for LTR activity and expressed relative to the shControl. Data is shown as mean and SD of three independent experiments. Significance was determined with an unpaired student’s T test. *p<0.05, **p<0.01, ***p<0.001.
(TIF)
