# Supplementary figures and images for: DYRK1A Controls HIV-1 Replication at a Transcriptional Level in an NFAT Dependent Manner
Source: PLoS One. 2015 Dec 7;10(12):e0144229. doi: 10.1371/journal.pone.0144229 (PMC4979971; doi:10.1371/journal.pone.0144229)

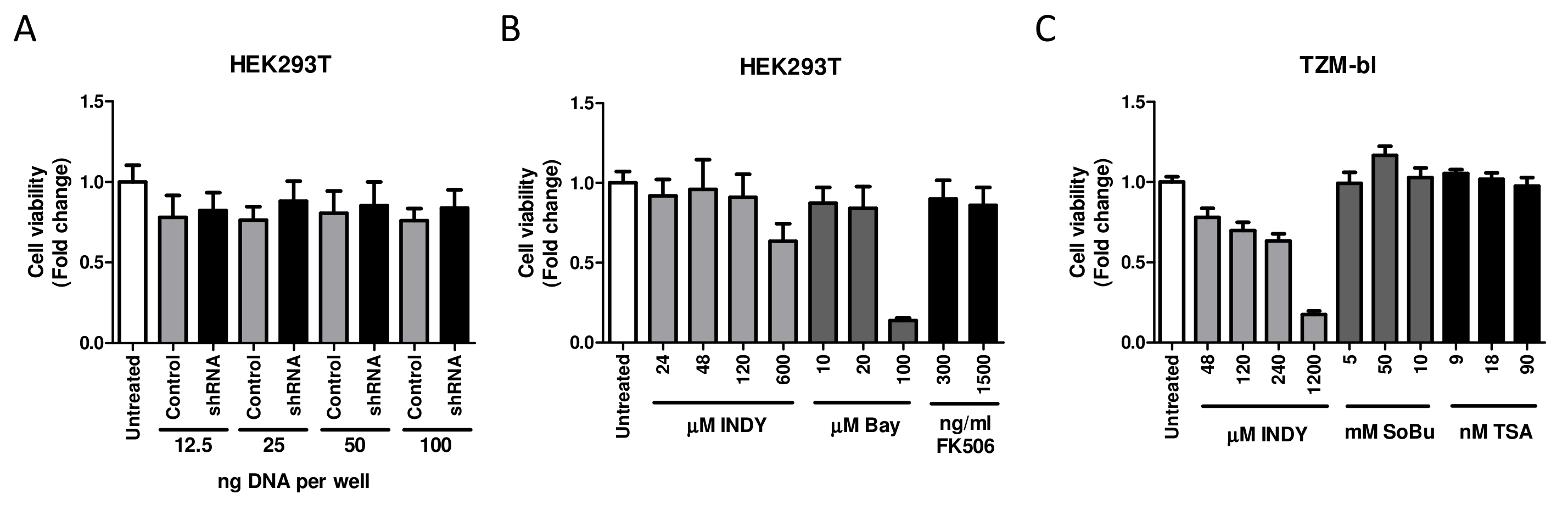

Supplement: S1 Fig — Effect of shRNAs (A) and inhibitors (B) on cell viability of HEK293T cells and (C) TZM-bl cells. Cell viability was assessed by MTT assay. Results are plotted as the mean and SD of at least two independent experiments and plotted as the fold change as compared to the untreated control cells. (TIF) [file pone.0144229.s001.tif]

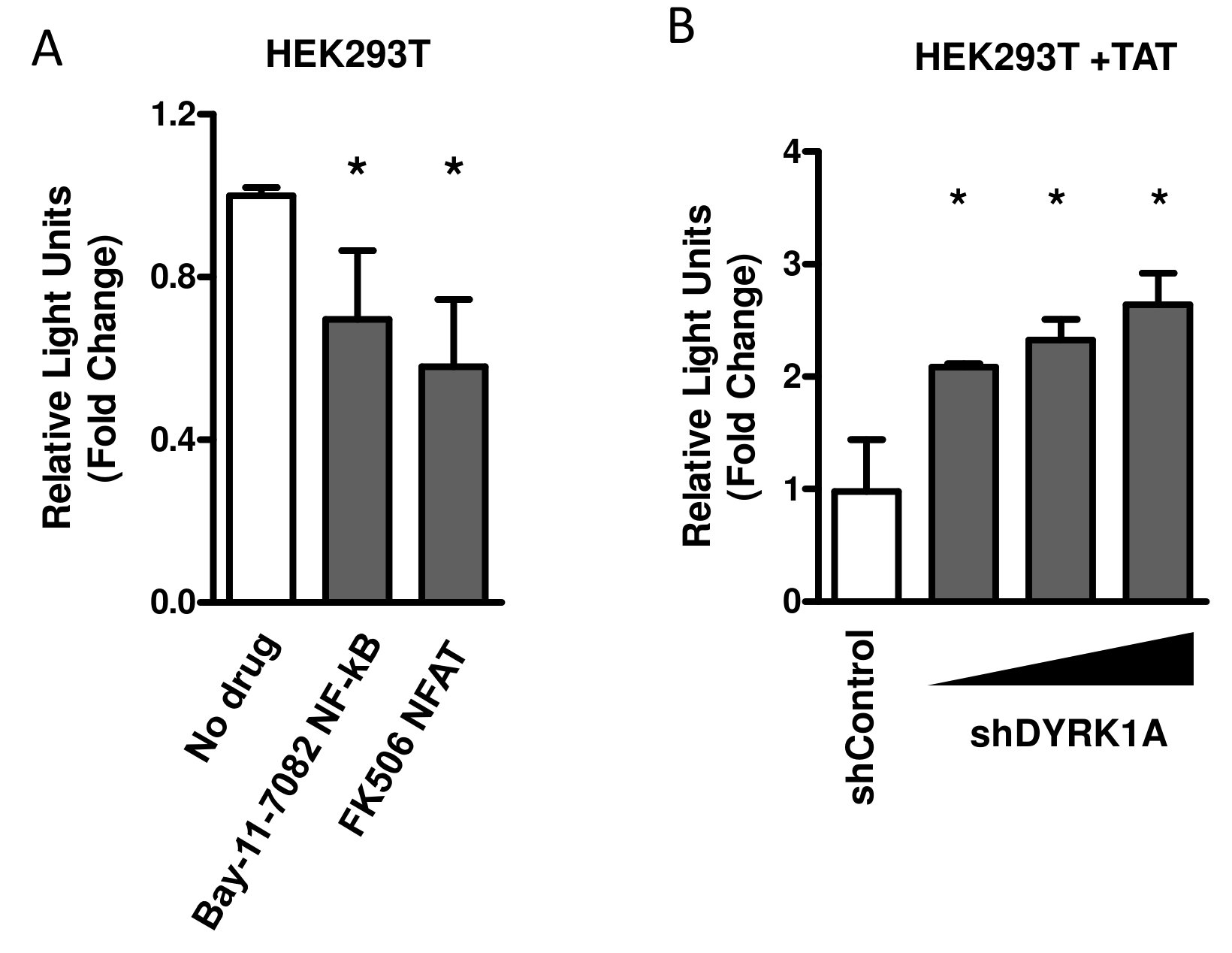

Supplement: S2 Fig — (A) The effect of NF-kb inhibitor Bay and NFAT inhibitor FK506 on basal LTR-driven luciferase expression in HEK293T cells. HEK293T cells were transfected in 96-wells plates with 5 ng of LTR-luciferase reporter construct and treated with 10 μm Bay or 300 ng/ml FK506 24 hours post transfection. Luciferase activity was analyzed 48-hours post-transfection as a measure for LTR activity and expressed relative to the No drug control. Data is shown as mean and SD of three independent experiments. (B) The effect of DYRK1A downregulation on LTR driven transcription in the presence of HIV-Tat was analyzed by co-transfection of HEK293T cells in 96-wells plates with 5 ng of LTR-luciferase reporter construct, 5 ng SV-Tat and 12.5 ng, 25 ng or 50 ng of shDYRK1A or the shControl vector. Luciferase activity was analyzed 48-hours post-transfection as a measure for LTR activity and expressed relative to the shControl. Data is shown as mean and SD of three independent experiments. Significance was determined with an unpaired student’s T test. *p<0.05, **p<0.01, ***p<0.001. (TIF) [file pone.0144229.s002.tif]
